# 

**Published:** 1898-02

**Authors:** 


### CONTENTS:

### SELECTIONS.

Article I.—The Standard of the National Board of Dental Examiners .......... 433

" II.—Economics of Dentition. By Dr. J. J. Madden .......... 441

" III.—Extracting Teeth. By Dr. F. C. M'Pherson .......... 446

" IV.—Formagen. By Dr. Abraham Konitz .......... 450

" V.—Drainage und Irrigation of the Antrum. By. E. L. Townsend, D. D., S .......... 452

" VI.—Tumors Resulting from Septic Pulp of Teeth. By G. Lennox Curtis, M. D .......... 454

" VII.—Treatment and Filling of Root Canals. By H. H. Sullivan, D. D. S .......... 458

" VIII.—Queer Things about Mankind .......... 464

" IX.—Prominent Causes of Failure in the Use of Electricity in Dentistry. By C. S. Neiswangey, M. D .......... 468

" X.—Urotropin. By J. A. Flexner, M. D .......... 472

### MONTHLY SUMMARY.

Gold Fillings Inserted while Cavity Filled with Saliva .......... 474

Rontgen Rays .......... 475

The Care of Vulcanizers .......... 475

Value of Meat Extracts .......... 477

Blood Stains .......... 477

Hints .......... 477

Experience .......... 479

Ingratitude .......... 480

